# Kisspeptin Restores Placental mTOR Signaling and Improves Glucose Homeostasis Mediators Disrupted by Maternal Hypothyroidism in Rats

**DOI:** 10.1111/apha.70188

**Published:** 2026-03-04

**Authors:** Bianca Reis Santos, Jeane Martinha dos Anjos Cordeiro, Luciano Cardoso Santos, Cleisla Souza Oliveira, Maria Clara Pascoal Santos Alvarez, Natália Panhoca Rodrigues, Jorge Lopez‐Tello, Amanda N. Sferruzzi‐Perri, Rogéria Serakides, Juneo Freitas Silva

**Affiliations:** ^1^ Centro de Microscopia Eletronica, Departamento de Ciencias Biologicas Universidade Estadual de Santa Cruz Ilheus Brazil; ^2^ Department of Physiology, Faculty of Medicine Autonomous University of Madrid Madrid Spain; ^3^ Centre for Trophoblast Research, Department of Physiology, Development and Neuroscience University of Cambridge Cambridge UK; ^4^ Departamento de Clinica e Cirurgia Veterinarias, Escola de Veterinaria Universidade Federal de Minas Gerais Belo Horizonte Brazil

**Keywords:** fetus, glucose, Kiss1, metabolism, PTU, thyroid

## Abstract

**Aim:**

Reduced placental mTOR signaling is associated with intrauterine growth restriction and impaired maternal and placental metabolism. Since maternal hypothyroidism induces intrauterine growth restriction, and maternal treatment with kisspeptin‐10 (Kp10) has been shown to improve feto‐placental development in hypothyroid rats, this study aimed to evaluate the effects of maternal hypothyroidism, with and without kisspeptin‐10 treatment, on maternal energy homeostasis and placental expression of mTOR and glucose metabolism mediators.

**Methods:**

Maternal hypothyroidism was induced by administration of propylthiouracil, and kisspeptin‐10 treatment began on gestational day 8.

**Results:**

Maternal hypothyroidism caused glucose intolerance, decreased insulin and HDL levels, reduced fetal and placental weights, and thinned the placental interhaemal barrier. It also increased INSRβ and AKT, while downregulating placental p‐mTOR/mTOR and Glut1. Although kisspeptin‐10 treatment did not improve maternal glucose homeostasis or prevent feto‐placental growth restriction, it attenuated maternal hypothyroidism‐induced placental Glut1 dysregulation, upregulated the IGF1/IGF1R axis, and restored placental AKT/mTOR expression.

**Conclusion:**

These findings suggest that kisspeptin‐10 treatment in hypothyroid pregnant rats improves placental mTOR signaling and glucose metabolism mediators, highlighting novel pathways through which kisspeptin may modulate placental physiology.

## Summary

- Hypothyroidism disrupts maternal and placental metabolism impairing nutrient sensing, fetal growth, and long‐term metabolic health.
- Kisspeptin restores placental mTOR signaling and glucose metabolism mediators in hypothyroid pregnant rats.
- Targeting kisspeptin signaling may represent a novel therapeutic strategy to mitigate pregnancy complications.

## 1 Introduction

The placenta plays a central endocrine role during pregnancy by regulating maternal metabolism and supporting fetal growth, particularly through adaptations in glucose homeostasis, including peripheral insulin resistance and increased basal glucose levels, which facilitate transplacental glucose transport [1, 2, 3, 4, 5]. This regulation is mediated by placental hormones such as placental lactogens (PL), insulin‐like growth factor 1 (IGF‐1), leptin, and prolactin, which modulate maternal insulin sensitivity and pancreatic β‐cell function via the placental–pancreatic axis [1, 4, 5, 6, 7, 8]. Disruption of these pathways has been implicated in gestational disorders such as gestational diabetes mellitus (GDM), preeclampsia, and intrauterine growth restriction (IUGR) [1, 2, 9].

At the molecular level, changes in placenta insulin signaling and metabolism also play a role in determining pregnancy outcomes and long‐term health. Alterations in the IRS/PI3K/mTOR signaling pathway in the placenta have been implicated in abnormal fetal growth and GDM [10, 11, 12]. The mTOR complex 1 (mTORC1), a key placental nutrient sensor, has been proposed as a mediator linking placental function to the programming of metabolic dysfunction and insulin resistance in adult offspring [11, 13, 14]. Furthermore, dysregulation of the IRS/PI3K/AKT/mTOR pathway is associated with structural and functional changes in the placenta that are linked to IUGR, obesity, GDM, and preeclampsia [11, 15, 16], highlighting mTOR as a potential therapeutic target for gestational disorders [12]. In addition, accumulating evidence indicates that placental mTOR signaling is sexually dimorphic, with male fetuses favoring growth‐promoting strategies and female fetuses prioritizing adaptive and reserve pathways [17], thereby contributing to differences in fetal growth trajectories and susceptibility to gestational complications [8].

Maternal hypothyroidism is a significant gestational metabolic disorder associated with placental dysfunction and IUGR [18, 19, 20]. However, its potential impact on placental mTOR signaling has not yet been investigated. It is also linked with impaired maternal glucose homeostasis and reduced placental hormones, including PL‐II [21, 22] and kisspeptin in hypothyroid rats [23]. Administration of kisspeptin‐10 in hypothyroid pregnant rats has been shown to improve the intrauterine environment by reducing inflammation and oxidative stress, and enhance fetal development [24, 25].

Although kisspeptin is primarily known for its role in regulating the hypothalamic–pituitary‐gonadal (HPG) axis [26, 27], it is now acknowledged as a key peptide for maintaining a healthy pregnancy [28, 29, 30]. Emerging evidence also indicates that kisspeptin contributes to glucose homeostasis by modulating insulin release and stimulating pancreatic β‐cell adaptation during pregnancy [31, 32, 33, 34]. Notably, oscillations in plasma and placental kisspeptin levels have been associated with GDM [33, 35, 36], suggesting that disrupted placental kisspeptin signaling may impair maternal glucose regulation and contribute to GDM pathogenesis signaling [6, 33, 34, 37]. In fact, exogenous kisspeptin‐10 administration in hypothyroid rats increases placental expression of placental growth factor (PlGF), IGF‐1, and glucose transporter 1 (GLUT1) [25], supporting its modulatory role in placental glucose homeostasis.

Despite these advances, significant gaps remain in our understanding of the placental molecular pathways through which kisspeptin exerts its metabolic effects during pregnancy. Notably, impaired placental mTOR signaling and reduced circulating kisspeptin levels have each been independently associated with intrauterine growth restriction (IUGR) and adverse pregnancy outcomes [14, 16, 28, 38]. However, to date, no studies have directly investigated the potential cross‐talk between kisspeptin signaling and placental mTOR–mediated metabolic regulation. Therefore, we hypothesized that maternal hypothyroidism disrupts placental mTOR signaling and glucose metabolism, and that kisspeptin‐10 treatment could restore these pathways, thereby improving maternal glucose homeostasis. Using a rat model, this study aimed to evaluate the effects of maternal hypothyroidism, with and without kisspeptin‐10 treatment, on maternal glucose homeostasis and placental expression of mTOR and key mediators of glucose metabolism.

## 2 Results

### 2.1 Maternal Kp10 Treatment Did Not Affect Glycemic Dysfunction, Reduced Plasma Insulin, and Decreased HDL Levels Induced by Hypothyroidism in Pregnant Rats

**FIGURE 1:**
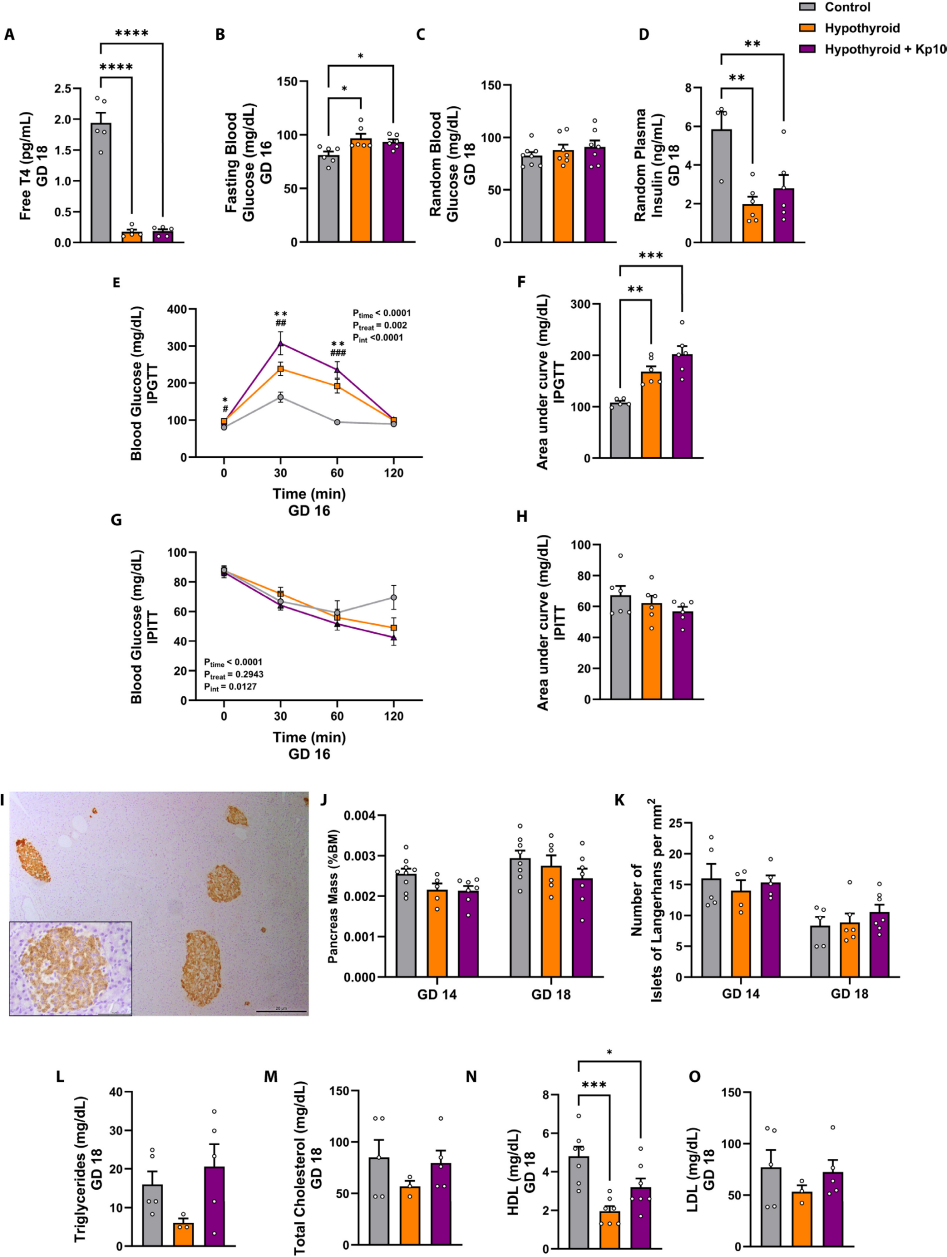
Maternal metabolic profile of control, hypothyroid, and Kisspeptin‐10 (Kp10)‐treated hypothyroid rats. (A) Maternal plasma level of free T4 at GD 18. (B) Fasting blood glucose at GD 16 (C) Random blood glucose at GD 18 (D) Random plasma insulin at GD 18. (E) Glycemic curve of IPGTT at GD 16. (F) Area under the IPGTT curve. (G) Glycemic curve of the insulin tolerance test at GD 16. (H) Area under the IPITT curve. (I) Representative photomicrograph of the pancreas with insulin immunolabeling (Polymer‐based detection system, Harris hematoxylin, Bar = 20 μm and 50 μm); (J) Pancreas mass relative to maternal body mass at GD 14 and 18. (K) Number of islets of Langerhans per mm^2^ at GD 14 and 18. (L–O) Maternal triglycerides, total cholesterol, HDL and LDL levels at GD 18. (A–O, except E and G: One‐way ANOVA *post hoc* SNK; E and G: Two‐way ANOVA *post hoc* SNK; Mean ± SEM). Significant differences are indicated by *p < 0.05, **p < 0.01, ***p < 0.001, ****p < 0.0001, except for E where *p < 0.05 control vs. Hypothyroid; **p < 0.01 control vs. Hypothyroid; ^#^ *p* < 0.05 control vs. *p* < 0.01; ^##^ *p* < 0.01 control vs. *p* < 0.01; ^###^ *p* < 0.001 control vs. Kp10. IPGTT, intraperitoneal glucose tolerance test; IPITT, intraperitoneal insulin tolerance test; GD, gestational day; HDL, high‐density lipoprotein; LDL, low‐density lipoprotein.

As MH impairs maternal glycemia [39], we first evaluated Kp10 effects on glucose homeostasis in hypothyroid pregnant rats. The induction of hypothyroidism was confirmed by significantly reduced plasma free T4 levels in the hypothyroid group compared to controls (*p* < 0.0001). Kp10 treatment did not affect free T4 plasma levels, which remained comparable to those in the hypothyroid group (*p* > 0.05; Figure 1A).

On GD 16, MH significantly increased fasting blood glucose levels compared to the control group (*p* < 0.05), while Kp10 treatment had no significant effect on this parameter (*p* > 0.05; Figure 1B). In contrast, no significant differences were observed in random blood glucose levels among the groups at GD 18 (*p* > 0.05; Figure 1C). However, MH significantly reduced random plasma insulin levels at GD 18 (*p* < 0.01), and this effect was not altered by maternal Kp10 treatment (*p* > 0.05; Figure 1D).

At GD 16, the IPGTT revealed that MH significantly elevated glucose levels at 0, 30, and 60 min compared to controls (*p* < 0.05; *p* < 0.01; Figure 1E), indicating impaired glucose intolerance. This was further supported by AUC analysis, which confirmed glucose intolerance in the MH group (*p* < 0.01; Figure 1F). Kp10 treatment did not ameliorate the glucose intolerance observed in hypothyroid dams, as glucose levels at 0, 30, and 60 min remained significantly elevated compared to controls (*p* < 0.05; *p* < 0.01; *p* < 0.001; Figure 1E,F). Regarding the IPITT, no significant differences in insulin sensitivity were observed among the groups (*p* > 0.05; Figure 1G,H).

Maternal pancreatic mass and insulin‐positive islets were quantified on GD 18 to complement glucose and insulin analyses. No significant differences were observed among groups (*p* > 0.05; Figure 1I–K).

Given the glucose intolerance in hypothyroid groups, lipid metabolism was assessed by measuring triglycerides, total cholesterol, and lipoprotein fractions, due to their close link with glucose metabolism in energy homeostasis [40]. Although no significant differences were found in plasma triglyceride, total cholesterol, or LDL concentrations among the groups (*p* > 0.05; Figure 1L,M,O), MH significantly reduced plasma HDL levels compared to controls (*p* < 0.05). Kp10 treatment did not reverse this reduction (*p* > 0.05; Figure 1N).

### 2.2 Kp10 Treatment Did Not Mitigate the Fetal Growth Restriction Caused by Hypothyroidism

**FIGURE 2:**
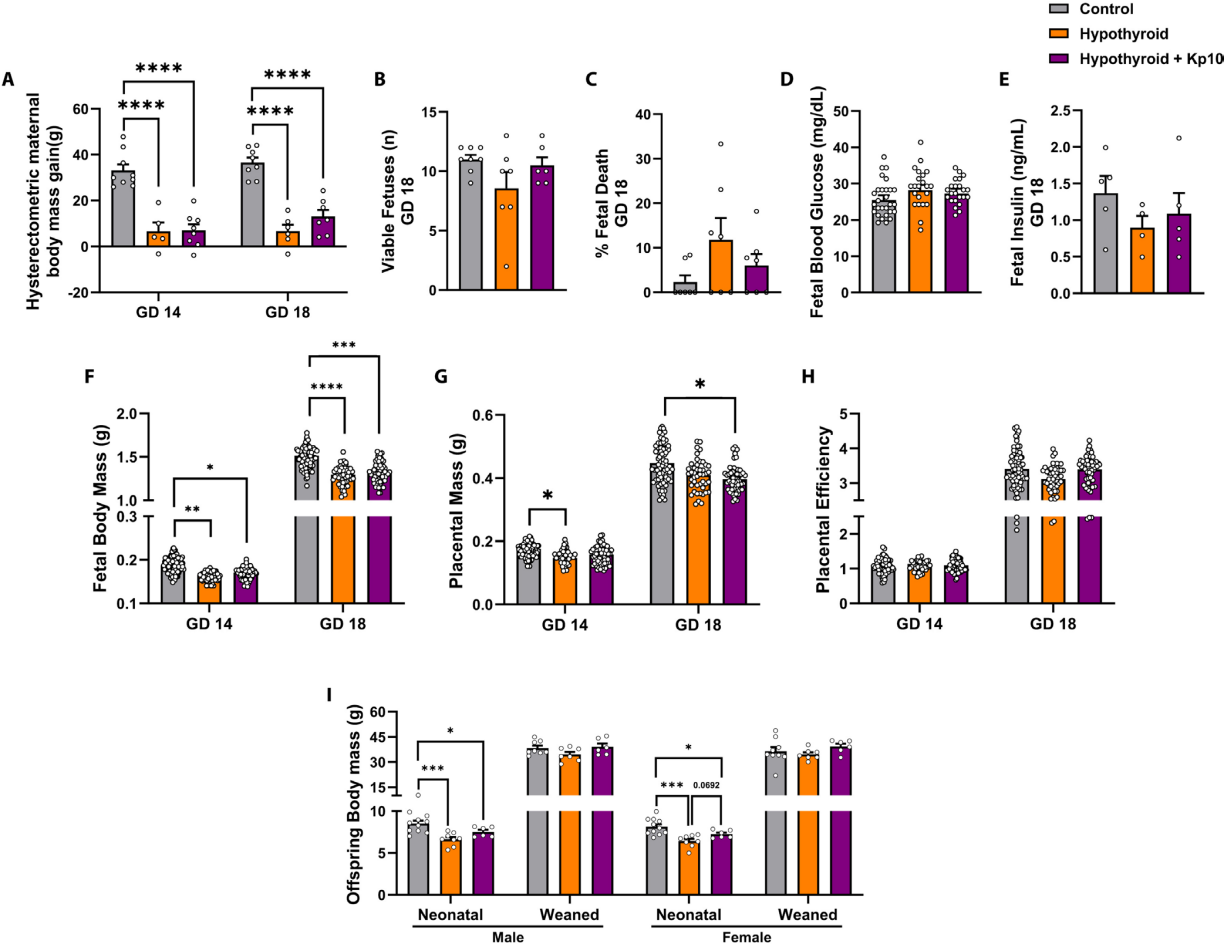
Maternal and offspring data from control, hypothyroid, and hypothyroid female rats treated with Kisspeptin‐10 (Kp10). (A) Maternal hysterectomised body mass gain at between GD 0 and GD 14 and GD 18. (B) Number of viable fetuses at GD 18. (C) Fetal death rate at GD 18. (D) Fetal blood glucose at GD 18. (E) Fetal plasma insulin at GD 18. (F) Fetal body mass at GD 14 and 18. (G) Placental mass at GD 14 and 18. (H) Placental efficiency calculated as the ratio of fetal to placental mass at GD 14 and 18. (I) Body mass of neonatal and weaned offspring. (A–C, H, I: One‐way ANOVA *post hoc* SNK; D–G: Linear mixed model *post hoc* Bonferroni; Mean ± SEM). Significant differences are indicated by *p < 0.05, **p < 0.01, ***p < 0.001, and ****p < 0.0001. In (D–G), graphs display the mean ± SEM and were analyzed using linear mixed model analysis. Individual fetal data points are overlaid on the graphs, prepared using Adobe Illustrator. GD, gestational day.

MH significantly reduced maternal body mass gain at both GD 14 and GD 18 compared to controls (*p* < 0.0001), and this effect was not alleviated by Kp10 treatment (*p* > 0.05; Figure 2A). Regarding fetal development, no significant differences were observed in the number of viable fetuses, fetal death rates, or fetal plasma glucose and insulin levels at GD18 (*p* > 0.05; Figure 2B–E). However, MH led to a significant reduction in fetal mass at both GD 14 and GD 18 (*p* < 0.01; *p* < 0.0001), and Kp10 treatment did not prevent this reduction (*p* > 0.05; Figure 2F).

At GD 14, MH significantly reduced placental mass compared to controls (*p* < 0.05), an effect that was partially mitigated by Kp10 treatment, as no significant difference was observed between the Kp10 treated MH group and controls (*p* > 0.05). However, by GD 18, MH alone no longer affected placental mass (*p* > 0.05), whereas Kp10 treatment of hypothyroid animals led to a significant reduction in placental mass compared to controls (*p* < 0.05; Figure 2G). No significant differences in placental efficiency were detected among the groups at either gestational age (*p* > 0.05; Figure 2H).

MH was also associated with a significant reduction in the body mass of both male and female neonatal offspring compared to controls (*p* < 0.001). Kp10 treatment showed a trend toward preventing this reduction in female neonates, though the difference did not reach statistical significance (*p =* 0.0692). No significant differences in the body mass of weaned offspring of either sex were observed among the groups (*p* > 0.05; Figure 2I).

### 2.3 Kp10 Treatment Prevents Glycogen Accumulation in the Junctional Zone Caused by Hypothyroidism

**FIGURE 3:**
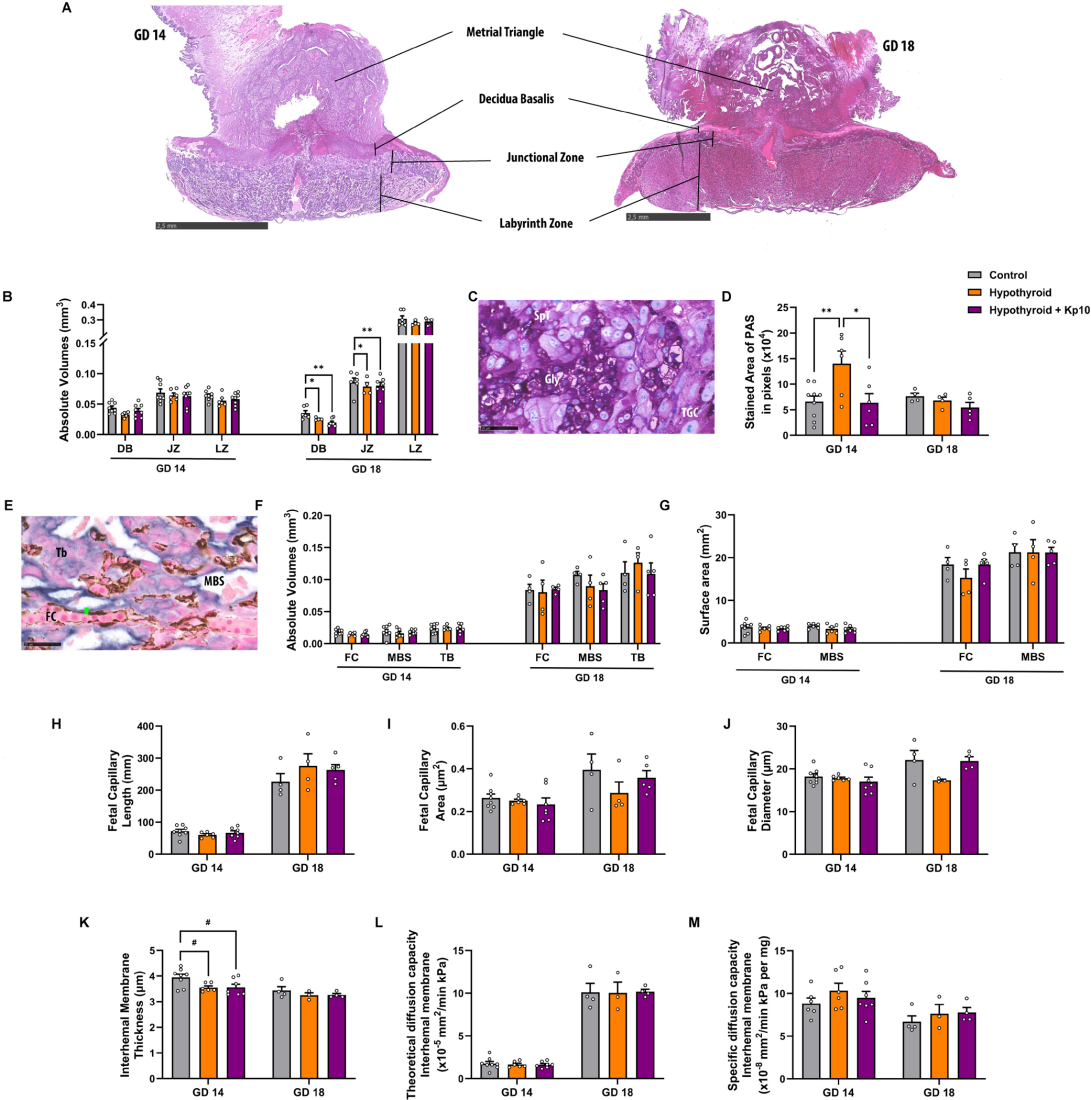
Assessment of placental morphology in control, hypothyroid, and Kisspeptin‐10 (Kp10)‐treated hypothyroid rats. (A) Photomicrographs of placentas at GD 14 (Hematoxylin & Eosin; Bar = 2.5 mm). (B) Absolute volume of the placental zones at GD 14 and 18. (C) Photomicrographs of PAS staining in the junctional zone (PAS; Fast Green; Bar = 50 μm). (D) Area in pixels stained by PAS at GD 14 and 18. (E) Photomicrographs of the double staining by cytokeratin + vimentin (Streptavidin‐biotin‐peroxidase; Alkaline Phosphatase‐BCIP/NBT; nuclear Fast Red; Bar = 20 μm) (F) Absolute volume of the compartments of the labyrinth zone at GD 14 and 18. (G) Surface area of the vasculature of the labyrinth area at GD 14 and 18. (H) Total fetal capillary length at GD 14 and 18. (I) Fetal capillary area at GD 14 and 18. (J) Fetal capillary diameter at GD 14 and 18. (K) Interhaemal membrane thickness at GD 14 and 18. (L) Theoretical diffusion capacity of the interhaemal membrane at GD 14 and 18. (M) Specific diffusion capacity of the interhaemal membrane at GD 14 and 18. (Mean ± SEM). Significant differences are indicated by *p < 0.05, **p < 0.01 for one‐way ANOVA followed by SNK test, and ^#^ *p* < 0.05 for Student's *t*‐test. BD, basal decidua; GD, gestational day; Gly, glycogen cells; Green bar, interhaemal membraneJZ, junctional zone; LZ, labyrinth zone; MVS, maternal vascular space; PAS, Periodic acid‐Schiff; SpT, spongiotrophoblast; TB, trophoblast; TGC, trophoblast giant cells.

Since changes in fetal growth may result from placental structural alterations, stereological analysis was performed [15]. At GD 14, no significant differences in placental layer volumes were observed among the groups (*p* > 0.05). However, at GD 18, MH significantly reduced the volumes of the basal decidua and junctional zone compared to controls (*p* < 0.05), whereas the labyrinth zone did not show a significant difference (*p* > 0.05). Kp10 treatment did not prevent these reductions in hypothyroid animals (*p* > 0.05; Figure 3A,B).

PAS staining (Figure 3C) revealed that glycogen accumulation in the junctional zone was significantly increased by MH at GD 14 (*p* < 0.01), and this effect was completely ameliorated by Kp10 treatment (*p* > 0.05). By GD 18, no significant differences in glycogen accumulation were observed among the groups (*p* > 0.05; Figure 3D).

Double‐label immunostaining for cytokeratin and vimentin (Figure 3E) showed no significant differences among groups in the absolute volumes or surface areas of fetal capillaries, maternal blood spaces and trophoblast (*p* > 0.05; Figure 3F,G). Similarly, there were no differences in fetal capillary length, area, or diameter (*p* > 0.05; Figure 3H–J). However, MH significantly thinned the interhaemal membrane compared to controls (*p* < 0.05), an effect not reverse by Kp10 treatment (*p* > 0.05; Figure 3K). No significant differences were found among the groups in theoretical diffusion capacity and specific interhaemal membrane diffusion (*p* > 0.05; Figure 3L,M).

### 2.4 Kp10 Treatment Attenuated the Placental Glut1 Dysregulation Caused by Hypothyroidism

**FIGURE 4:**
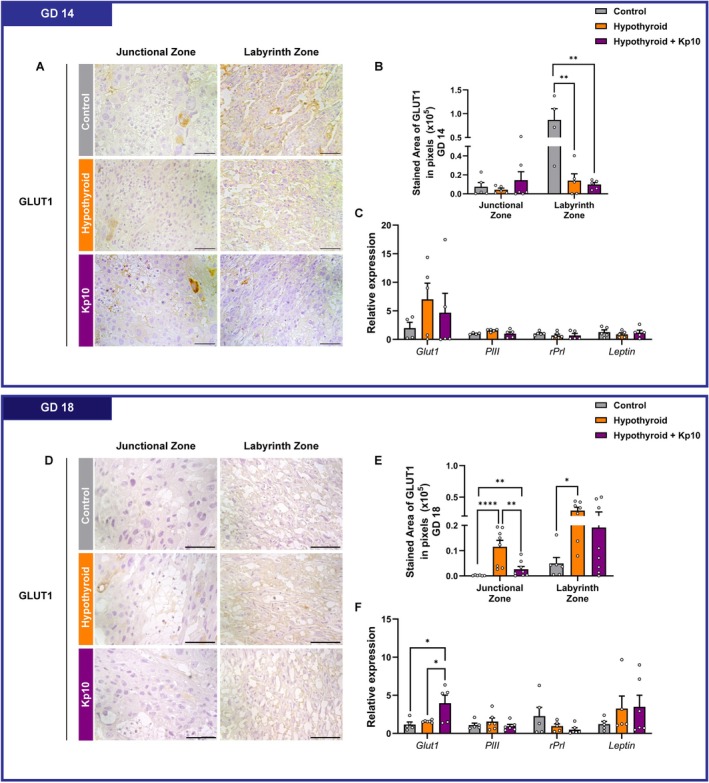
Assessment of GLUT1 expression and hormonal factors in placental tissue from control, hypothyroid, and Kisspeptin‐10 (Kp10)‐treated hypothyroid rats. (A) Photomicrographs of GLUT1 immunolabeling in the placenta at GD 14 (Polymer‐based detection system; Harris hematoxylin). (B) Area in pixels of GLUT1 immunolabeling at GD 14. (C) Gene expression of *Glut1*, *PlII*, *rPrl and Leptin* at GD 14. (D) Photomicrographs of GLUT1 immunolabeling in the placenta at GD 18 (Polymer‐based detection system; Harris hematoxylin). (E) Area in pixels of GLUT1 immunolabeling at GD 18. (F) Gene expression of *Glut1*, *PlII*, *rPrl and Leptin* at GD 18 (Mean ± SEM). Significant differences are indicated by *p < 0.05, **p < 0.01, ****p < 0.0001, using one‐way ANOVA followed by SNK post hoc test. GD, gestational day. Bar = 50 μm.

Given the essential role of placental glucose transfer in fetal growth [5], we assessed the expression of glucose transporter GLUT1, along with placental hormones involved in maternal metabolic adaptations that support nutrient supply, including PL‐II, rPRL, and Leptin. GLUT1 immunostaining was most prominent in the labyrinth zone, particularly at GD 14 (Figure 4A,D). At GD 14, MH significantly reduced GLUT1 immunostaining in the labyrinth zone compared to controls (*p* < 0.01), and this reduction was not prevented by Kp10 treatment (*p* > 0.05). No significant differences were observed in the junctional zone at this gestational age (*p* > 0.05; Figure 4B). At the transcript level, no significant differences in the expression of *Glut1*, *PlII*, *rPrl*, or *Leptin* were detected among groups when whole placentas were analyzed (*p* > 0.05; Figure 4C).

By GD 18, MH significantly increased GLUT1 immunostaining in both the junctional and labyrinth zones compared to controls (*p* < 0.0001, *p* < 0.05). Kp10 treatment prevented the increase in the junctional zone (*p* < 0.01), although GLUT1 levels remained moderately but significantly elevated compared to controls (*p* < 0.01). In the labyrinth zone, Kp10 treatment partially prevented the increase, resulting in no significant difference compared to controls (*p* > 0.05; Figure 4E). Interestingly, while MH alone had no significant effect on *Glut1* mRNA levels, Kp10 treatment increased significantly increased *Glut1* expression compared to both control and hypothyroid groups (*p* < 0.05). No differences were observed in the expression of *PlII*, *rPrl* or *Leptin* transcripts among the groups (*p* > 0.05; Figure 4F).

### 2.5 Maternal Hypothyroidism Increased Placental INSRβ Expression, While Kp10 Upregulated IGF1/IGF1R Expression

**FIGURE 5:**
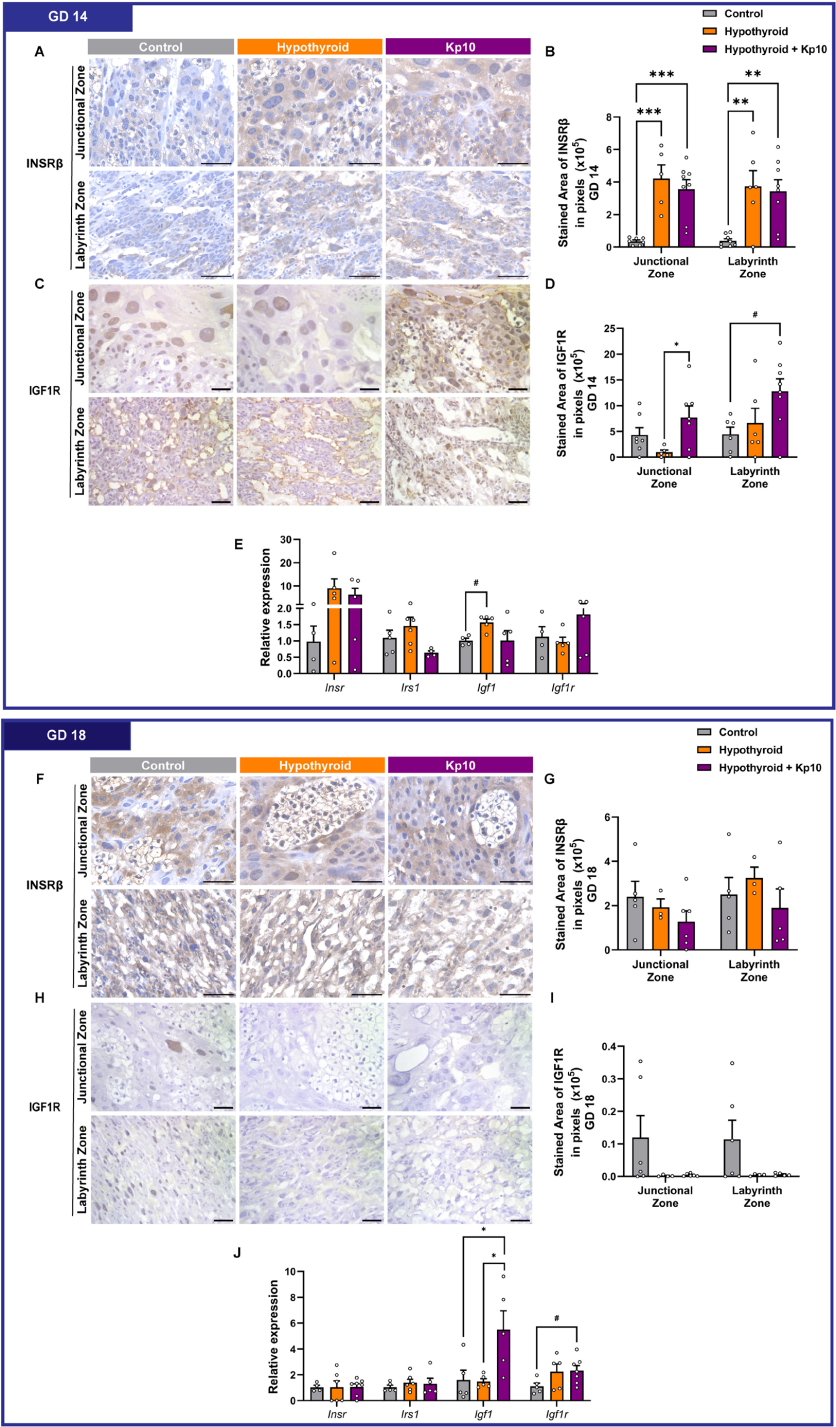
Assessment of INSR/IGF1/IGF1R system expression in placental tissue from control, hypothyroid, and Kisspeptin‐10 (Kp10)‐treated hypothyroid rats. (A) Photomicrographs of INSRβ immunolabeling in the placenta at GD 14 (Polymer‐based detection system; Harris hematoxylin). (B) Area in pixels of INSRβ immunolabeling at GD 14. (C) Photomicrographs of IGF1R immunolabeling in the placenta at GD 14 (Polymer‐based detection system; Harris hematoxylin). (D) Area in pixels of IGF1R of immunolabeling at GD14. (E) Gene expression of *Insr*, *Irs1*, *Igf1* and *Igf1r* at GD 14. (F) Photomicrographs of INSRβ immunolabeling in the placenta at GD 18 (Polymer‐based detection system; Harris hematoxylin). (G) Area in pixels of INSRβ immunolabeling at GD 18. (H) Photomicrographs of IGF1R immunolabeling in the placenta at GD 18 (Polymer‐based detection system; Harris hematoxylin). (I) Area in pixels of IGF1R immunolabeling at GD 18. (J) Gene expression of *Insr*, *Irs1*, *Igf1* and *Igf1r* at GD 18. (Mean ± SEM). Significant differences are indicated by *p < 0.05, **p < 0.01, **p < 0.001, ****p < 0.0001 for one‐way ANOVA followed by SNK post hoc test; ^#^ *p* < 0.05 for Student's *t*‐test. GD, Gestational Day. Bar = 50 μm.

Since placental GLUT1 expression was dysregulated in hypothyroid animals, we evaluated the INSR/IGF1/IGF1R system, crucial for growth, metabolic signaling, and fetal‐placental development [41, 42]. INSRβ immunostaining in the placenta exhibited heterogeneous cytoplasmic distribution, with prominent labeling in spongiotrophoblasts and giant cells, particularly at GD 14 (Figure 5A,F). IGF1R immunostaining was predominantly nuclear in the junctional zone, while both nuclear and cytoplasmic staining were observed in the labyrinth zone. A marked reduction in IGF1R was noted at GD 18 compared to GD 14 (Figure 5C,H).

At GD 14, MH significantly increased INSRβ labeling in both placental zones compared to controls (*p* < 0.001, *p* < 0.01), and this upregulation was not altered by Kp10 treatment (*p* > 0.05; Figure 5B). Although no significant differences were observed in IGF1R expression between MH and control groups (*p* > 0.05), Kp10 treatment significantly increased IGF1R immunostaining in the junctional and labyrinth zones compared to hypothyroid and control groups, respectively (*p* < 0.05; Figure 5D). At the transcript level (whole placenta analysis), MH significantly upregulated *Igf1* expression compared to controls (*p* < 0.05). This effect was mitigated by Kp10 treatment, which restored Igf1 levels to those similar to the control group (*p* > 0.05). No significant differences were observed in the mRNA expression levels of *Insr*, *Igf1r*, or *Irs1* among groups at GD 14 (*p* > 0.05; Figure 5E).

At GD 18, no significant differences were observed in the area of INSRβ and IGF1R immunostaining among groups (*p* > 0.05; Figure 5G,I). However, a qualitative reduction in IGF1R staining was noted in both hypothyroid groups (untreated and treated), with expression ranging from weak to absent compared to controls. At the transcript level, while MH did not affect *Igf1* or *Igf1r* expression, Kp10 treatment significantly increased their expression relative to the control group (*p* < 0.05) and/or MH groups (*p* < 0.05). No significant differences were observed in *Insr* and *Irs1* mRNA levels among groups (*p* > 0.05; Figure 5J).

### 2.6 Maternal Kp10 Treatment Reversed Placental AKT/mTOR Dysregulation Induced by Hypothyroidism at GD 14

**FIGURE 6:**
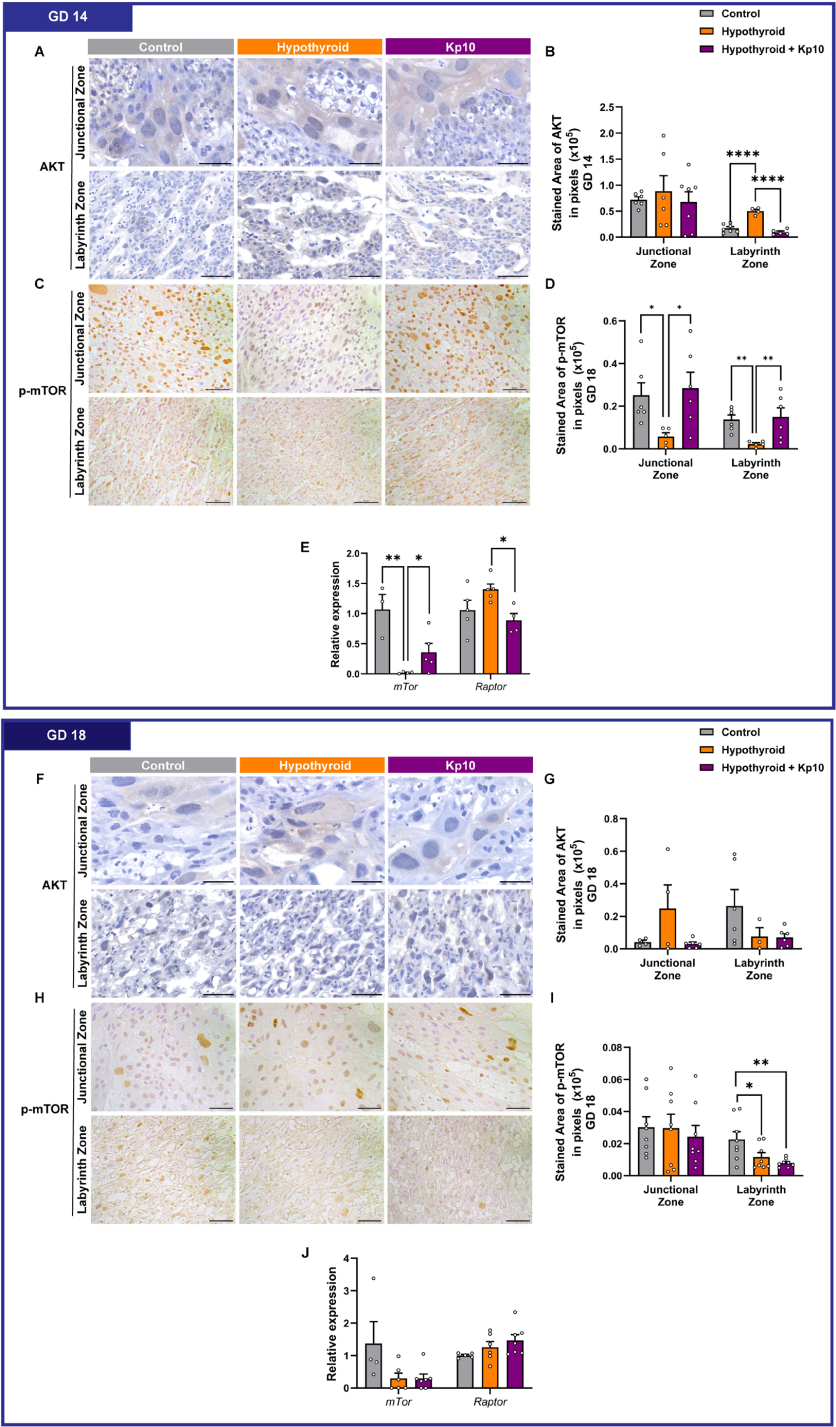
Assessment of AKT/mTOR signaling in placental tissue from control, hypothyroid, and Kisspeptin‐10 (Kp10)‐treated hypothyroid rats. (A) Photomicrographs of AKT immunolabeling in the placenta at GD 14 (Polymer‐based detection system; Harris hematoxylin). (B) Area in pixels of AKT immunolabeling at GD 14. (C) Photomicrographs of p‐mTOR immunolabeling in the placenta at GD 14 (Polymer‐based detection system; Harris hematoxylin). (D) Area in pixels of p‐mTOR immunolabeling at GD 14. (E) Gene expression of *mTor* and *Raptor* at GD 14. (F) Photomicrographs of AKT immunolabeling in the placenta at GD 18 (Polymer‐based detection system; Harris hematoxylin). (G) Area in pixels of AKT immunolabeling at GD 18. (H) Photomicrographs of p‐mTOR immunolabeling in the placenta at GD 18 (Polymer‐based detection system; Harris hematoxylin). (I) Area in pixels of p‐mTOR immunolabeling at GD 18. (J) Gene expression of *mTor and Raptor* at GD 18. (Mean ± SEM). Significant differences are indicated by *p < 0.05, **p < 0.01, and ****p < 0.0001 for one‐way ANOVA followed by SNK post hoc test. GD, gestational day. Bar = 50 μm.

Since mTOR signaling is closely linked to glucose metabolism and functions as a key nutrient sensor in the placenta [14], we evaluated components of the mTOR signaling pathway, namely AKT, p‐mTOR/*mTOR*, and *Raptor*. AKT immunostaining was cytoplasmic and heterogeneous and primarily localized to giant cells and labyrinth trophoblasts, with higher expression at GD 14 compared to GD 18 (Figure 6A,F).

At GD 14, MH significantly increased AKT immunostaining in the labyrinth zone compared to controls (*p* < 0.0001), an effect that was ameliorated by Kp10 treatment, restoring staining area to control levels (*p* > 0.05). No significant differences were found in AKT immunostaining within the junctional zone across groups (*p* > 0.05; Figure 6B).

Activation of mTOR, as assessed by p‐mTOR immunostaining, revealed intense and homogeneous nuclear labeling throughout the placenta at GD 14. MH significantly reduced expression in both zones (Figure 6C,H; *p* < 0.05, *p* < 0.01), an effect that was fully prevented by Kp10 treatment (*p* > 0.05 versus controls; Figure 6D). In line with these findings, placental *mTOR* mRNA expression was also reduced in the MH group compared to controls (*p* < 0.05), and Kp10 treatment restored *mTOR* transcript levels to those seen in controls (*p* > 0.05; Figure 6E). While MH did not significantly alter *Raptor* expression, Kp10 treatment reduced *Raptor* mRNA in hypothyroid animals (*p* < 0.05), aligning expression levels with the control group (*p* > 0.05; Figure 6E).

At GD 18, no significant differences in AKT immunostaining were observed among group (*p* > 0.05; Figure 6F). However, MH significantly decreased p‐mTOR immunostaining in the labyrinth zone compared to controls (*p* < 0.05), and this reduction was not reversed by Kp10 treatment (*p* > 0.05; Figure 6I). No significant differences were detected in p‐mTOR immunostaining within the junctional zone or in placental *mTor* and *Raptor* mRNA expression at this gestational age (*p* > 0.05; Figure 6I,J).

## 3 Discussion

This study demonstrates that glucose dysregulation and feto‐placental restriction induced by maternal hypothyroidism in pregnant rats are associated with altered placental expression of GLUT1 and INSRβ, as well as disrupted AKT/mTOR signaling. Notably, maternal Kp10 treatment improved GLUT1 expression, normalized AKT/mTOR signaling, while also upregulating the IGF1/IGF1R axis. These findings suggest novel mechanisms through which kisspeptin improves fetal development and the intrauterine environment of hypothyroid conditions [24, 25].

Maternal hypothyroidism led to elevated fasting blood glucose, impaired glucose tolerance, and reduced plasma insulin and HDL levels; metabolic alterations consistent with those observed in pregnant rats treated with the antithyroid drug methimazole [21], and in pregnant women with hypothyroidism [43]. Interestingly, the reduction in circulating insulin was not accompanied by a decrease in pancreatic mass or insulin‐positive islets, suggesting impaired insulin secretion rather than pancreatic islet loss in hypothyroid pregnant rats. Supporting this, Kent et al. also reported no changes in pancreatic mass and β‐cell mass under conditions of severe hypothyroidism during pregnancy in rats. However, the same study showed decreased *Glut4* expression and reduced insulin sensitivity mediators in the skeletal muscle and white adipose tissue of the animals [21]. These alterations, together with the reduced plasma insulin levels observed, may impair glucose uptake by peripheral tissues and account for the increase in circulating plasma glucose levels observed in the present study.

During pregnancy, maternal metabolism undergoes adaptations driven by placental hormones, including the development of a physiological state of insulin resistance that directs glucose to the fetus for growth [5]. Therefore, the impaired development of this insulin‐resistant environment may contribute to the glycemic dysfunction observed in hypothyroid pregnant rats, since non‐pregnant hypothyroid rats typically do not exhibit glucose intolerance [21]. Given that maternal glycemic disturbances can compromise fetal nutrient supply [8, 44, 45], these alterations may contribute to the feto‐placental restriction and reduced neonatal body mass observed in this study.

In contrast, Kp10 treatment failed to improve the glycemic dysfunction induced by hypothyroidism in pregnant rats. While several studies have demonstrated the beneficial effects of kisspeptin on insulin secretion, glucose homeostasis, and pancreatic β‐cell adaptive mechanisms during pregnancy [31, 33, 34, 46, 47, 48, 49, 50, 51, 52], its effects appear to be context dependent. In rats, kisspeptin administration has been shown to stimulate insulin secretion without affecting blood glucose levels [31]. Previous in vitro studies using mouse pancreatic islets have further shown that kisspeptin‐induced insulin secretion occurs only in the presence of glucose stimulation [31, 51]. In non‐pregnant mice, prolonged Kp10 exposure improves glycemia during an IPGTT, whereas kisspeptin antagonist (Kp234) administration or pancreatic *Kiss1r* gene deletion impairs glucose tolerance at GD 16 [33].

Given the short plasma half‐life of Kp10 (~4 min) [53, 54] and its rapid, glucose‐dependent effects on insulin secretion [31, 51], we hypothesized that its metabolic actions are transient. Thus, the lack of improvement in glucose metabolism in our Kp10‐treated animals may be explained by the 4‐h interval between Kp10 administration and the IPGTT. Therefore, it is plausible that Kp10 exerted effects within the first 30 min after the glucose challenge, which were not captured due to our experimental timing. Further studies are needed to test this hypothesis directly.

The reduction in fetal and placental mass observed in hypothyroid female rats, along with decreased placental zone dimensions and increased glycogen cell population in the junctional zone, is an established feature of maternal hypothyroidism in rats [23, 25, 55, 56]. However, the observed reduction in placental interhaemal barrier thickness at GD 14 is a unique and novel finding. This morphological alteration may represent an adaptive mechanism by which the placenta attempts to enhance substrate transfer to the growth‐restricted fetus, given that interhaemal barrier thickness is inversely proportional to diffusion capacity [57]. A similar adaptation is observed in placentas from experimental hypoxia models in mice [58], and previous studies have also suggested that maternal hypothyroidism is associated with placental hypoxia [25, 59].

Although Kp10 treatment did not restore feto‐placental growth or normalize the reduced interhaemal barrier thickness in hypothyroid female rats, it did normalize the glycogen cell population in the junctional zone, consistent with findings from our previous study [25]. Additionally, Kp10 showed a trend toward improving body mass in female neonatal offspring. These effects may reflect enhanced placental metabolic function. The junctional zone is a site for glycogen storage, which can be mobilized for fetal growth. In addition, it is the primary placental layer responsible for producing placental hormones critical for fetal and postnatal development [4, 60].

Our findings also demonstrated that maternal hypothyroidism disrupted placental GLUT1 protein expression, with a decrease at GD 14, when fetal growth begins to exponentially [61]. We also found that GLUT1 increased expression was increased at GD 18. Dysregulation of GLUT1 has been reported in various gestational disorders associated with IUGR, including models of iatrogenic hyperadrenocorticism [62] and hypothyroidism in female rats [39], as well as in placentas from women with preeclampsia [63].

The increased GLUT1 protein expression observed in late pregnancy in hypothyroid female rats may represent a compensatory response to the reduced GLUT1 levels seen at GD 14, potentially ensuring sufficient glucose supply for fetal development. Similarly, the upregulation of the insulin receptor INSRβ and *Igf1*, along with their common downstream effector AKT, may also reflect compensatory mechanisms to enhance placental glucose uptake, since AKT also stimulates this uptake [64, 65]. It is noteworthy that although the reduction in IGF1R immunostaining in hypothyroid animals was not statistically significant, the visible decrease may contribute to the observed upregulation of *Igf1* expression; a possibility that requires further studies.

Since mTOR is considered a nutrient oscillation sensor in the placenta [14, 15], its reduced expression in placentas from hypothyroid rats may reflect a cellular response to perceived nutrient restriction. This interpretation is consistent with the decreased GLUT1/*Glut1* and amino acid availability, which has previously been reported in pregnant hypothyroid rats [39]. Similarly, genetic ablation of mTOR in the placenta [11] or trophoblast [12] in mice results in reduced fetal weight [11, 12] and a leftward shift in the fetal weight distribution curve [12]. Furthermore, placental mTOR downregulation is a hallmark of gestational disorders associated with IUGR, as demonstrated in rat models of GDM [66] and in placentas from women with preeclampsia and IUGR [16, 67]. However, this is the first study to demonstrate reduced placental mTOR expression in maternal hypothyroidism. The increased AKT expression in hypothyroid animals may also represent a compensatory mechanism for reduced mTOR levels, as AKT activation typically precedes mTOR activation following insulin and IGF1 signaling [68, 69, 70].

Interestingly, maternal Kp10 treatment restored placental mTOR expression in hypothyroid rats at GD 14. Although more studies are needed to confirm the mechanistic link, an in vitro study has demonstrated that Kp10 can activate the mTOR pathway in bovine mammary epithelial cells [71]. Collectively, these studies indicate that kisspeptin may exert beneficial effects on placental function, at least in part, by positively modulating the mTOR signaling pathway.

However, in addition to the possibility of direct kisspeptin regulation of the mTOR pathway, an indirect regulation may also be involved. Oxidative stress is known to inhibit mTOR signaling [70], and previous studies have demonstrated the antioxidant potential of Kp10 in placental dysfunction with maternal hypothyroidism in rats [24, 25, 59]. Notably, Kp10 treatment also increased placental IGF1R expression at GD 14, as well as *Igf1* and *Igf1r* transcripts at GD 18. These changes may reflect the observed upregulation of mTOR, given that IGF1 and IGF1R are upstream regulators of this pathway [70, 72], and are associated with GLUT1 expression [64]. This mechanistic relationship also supports the increased *Glut1* expression observed in the placentas of the Kp10‐treated animals in the present study.

Although the upregulation of mTOR and *Igf1*/IGF1r/*Igf1r* signaling induced by Kp10 treatment was insufficient to reverse the reductions in fetal mass and placental morphological alterations, these molecular adaptations may help mitigate the damage caused by maternal hypothyroidism. Indeed, previous studies have shown that Kp10 alleviates oxidative stress [25] and inhibits pyroptosis [24] in the placentas of hypothyroid rats.

It is also noteworthy that mTOR signaling participates in mitochondrial function and cellular stress responses by coordinating mitochondrial protein translation in response to metabolic stimuli, thereby ensuring efficient energy production [73]. mTOR regulates the expression of key transcription factors such as PGC1 (peroxisome proliferator‐activated receptor gamma coactivator‐1), a central regulator of mitochondrial biogenesis and function, as well as HIF1α (hypoxia‐inducible factor 1 alpha) and ATF4 (activating transcription factor 4), which are critical for glycolysis, metabolic homeostasis, and redox signaling [70, 73]. Therefore, future studies should investigate mitochondrial homeostasis in maternal hypothyroidism and explore how kisspeptin treatment may modulate placental mitochondrial metabolism.

It is important to note that several mediators showed significant mRNA changes that were not always reflected in protein immunostaining (e.g., GLUT1, INSR, IGF1R, and mTOR). The relationship between mRNA and protein expression is complex, non‐linear, and gene‐ and context‐dependent [74, 75]. RT‐qPCR was performed on whole placental homogenates (junctional + labyrinth zones), whereas immunohistochemistry provides spatially resolved protein detection, which may partly explain these discrepancies. Additionally, mRNA and protein differ in synthesis and degradation kinetics: RT‐qPCR captures a snapshot of transcriptional activity, whereas protein levels may reflect cumulative expression over a longer period [75]. These considerations underscore that gene expression and protein levels are complementary, not necessarily proportional.

Despite the strength of the findings, our work has several limitations. The lack of placental sexing limited the ability to assess the potential role of fetal sex on the response to maternal hypothyroidism and the effects of kisspeptin. Additionally, immunostaining analysis could be corroborated by additional techniques like western blotting. Furthermore, measuring circulating kisspeptin levels would be valuable, as most human studies on kisspeptin in gestational disorders report alterations in plasma concentrations [33, 35, 36]. However, a major limitation is the current lack of a validated and reliable commercial assay for measuring plasma kisspeptin in rats.

Collectively, our findings demonstrate that maternal hypothyroidism‐induced glycemic dysfunction and IUGR in rats are associated with reduced placental AKT/mTOR signaling and dysregulated GLUT1/INSRβ expression. In addition, Kp10 treatment attenuated these alterations and positively modulated placental IGF1/IGF1R expression. To our knowledge, this is the first study to evaluate IGF1/mTOR signaling pathway in the placenta of hypothyroid rats and to identify novel mechanisms of kisspeptin action which may influence placental physiology.

## 4 Materials and Methods

### 4.1 Animals

Wistar rats were obtained from the Animal Breeding, Maintenance and Experimentation Laboratory (LaBIO), State University of Santa Cruz (Uesc). They were housed in groups (5–6 animals/cage) under controlled temperature (22°C ± 2°C) and light–dark cycles (12:12 h), with *ad libitum* access to water and commercial feed. All procedures were approved by Uesc Animal Care and Use Committee (Protocol CEUA N°. 026/22).

### 4.2 Experimental Design

Virgin female rats (210 ± 10 g) were randomized into three groups: control (*n* = 28), hypothyroid (*n* = 21), and hypothyroid treated with Kp10 (Kp10; *n* = 21). Hypothyroidism was induced with 6‐propyl‐2‐thiouracil (PTU; 4 mg/kg/day, oral gavage; Sigma‐Aldrich), and Kp10 treatment (8 μg/kg/day, i.p.; Tocris Bioscience) was initiated on gestational day (GD) 8, as described by [25].

Animals were euthanized at GD14 and GD18 (6–9 per group), corresponding to completion of placentation [76] and peak maternal pancreatic mass [7], respectively. Animals euthanized at GD18 underwent intraperitoneal glucose and insulin tolerance tests on GD16. Additional animals (6–11 per group) were allowed to deliver; litters were standardized to eight pups, and neonatal (3 days post‐birth) and weaning (21 days post‐birth) body mass were recorded.

### 4.3 Intraperitoneal Glucose Tolerance Test (IPGTT) and Intraperitoneal Insulin Tolerance Test (IPITT)

For the IPGTT, baseline blood glucose was measured after a 6‐h fast (11:00–12:00 h), followed by intraperitoneal glucose (2 g/kg) with measurements at 30, 60, and 120 min. After refeeding and a 1‐h fast, baseline glycemia was reassessed for the IPITT, and insulin (0.75 IU/kg) was administered intraperitoneally, with glucose measured at 30, 60, and 120 min. Tail‐tip samples were used with a glucometer (Accu‐Check Performa, Roche). IPITT was performed the same day to minimize stress, as no differences were observed compared with separate‐day tests (1‐week interval) [77]. Area under the curve (AUC) was calculated by the trapezoidal rule [78].

### 4.4 Necropsy and Sample Collection

Euthanasia was performed in the morning (9:00–12:00). Prior to euthanasia, maternal body weight was measured to determine gestational weight gain. Blood glucose levels were measured using a glucometer from maternal tail‐tip and fetal cervical blood. At GD 14 and 18, dams and fetuses were euthanized by decapitation, and blood was collected from the cervical region in heparinized tubes, centrifuged (3000 rpm, 20 min), and plasma stored at −20°C.

During necropsy, the gravid uterus and maternal pancreas were collected and weighed. Placentas and fetuses were dissected and weighed, viable and non‐viable fetuses (dead and resorbed) were counted. Viable fetuses displayed a healthy pink coloration, whereas non‐viable fetuses were typically small, hemorrhagic and/or emaciated and pale, hysterectomized maternal body mass gain was calculated. Two placentas from viable fetuses per litter were placed in TRIzol, snap‐frozen, and stored at −80°C for real‐time polymerase chain reaction (RT‐qPCR) analysis. Two implantation sites (placenta + decidua) and maternal pancreas samples per litter were fixed in 4% paraformaldehyde (4°C, 24 h), paraffin‐embedded, sectioned (4 μm), and mounted for histological and immunohistochemical analyses. Immunostaining was performed on silanized StarFrost slides (Knittel Glass). All procedures followed Santos et al. (2022b).

### 4.5 Analysis of Hormones and Metabolites in the Circulation

Free T4 (IMMULITE, Siemens) and plasma insulin (EZRMI‐13 K; EMD Millipore) were measured by ELISA (sensitivities: 0.4 ng/dL and 0.1 ng/mL). Plasma triglycerides (K117; Bioclin), total cholesterol (100/280–500; VIDA Biotecnologia), and HDL (K071; Bioclin) were quantified on a BIO‐2000 semi‐automated analyzer. All procedures were performed according to manufacturers' instructions. LDL was calculated via the Friedewald formula.

### 4.6 Histomorphometric Analysis of the Placenta

Placental histomorphometry was performed on Hematoxylin–Eosin, Periodic Acid‐Schiff (PAS)‐Fast Green, and cytokeratin–vimentin double‐labeled sections. Junctional and labyrinth zone areas were quantified using NDP.view2 software (Hamamatsu Photonics K.K., Hamamatsu). Glycogen accumulation in junctional zone glycogen cells was quantified on PAS‐stained sections using ImageJ software (U.S. National Institutes of Health).

In the labyrinth zone, dual immunostaining for cytokeratin and vimentin was performed to identify trophoblasts and fetal capillaries, respectively, following a modified protocol from De Clercq et al. Sections were deparaffinized, rehydrated, and endogenous peroxidase blocked (3% H_2_O_2_, 10 min). Antigen retrieval for vimentin was performed in citrate buffer (pH 6, 80°C, 30 min, plus 10 min at room temperature). Sections were blocked (10% goat serum in blocking buffer, 15 min) and incubated overnight at 4°C with anti‐vimentin (ab92547, 1:200, Abcam). The next day, sections were incubated at room temperature (1.5 h), followed by goat secondary antibody (ab6720, 1:1000, Abcam, 1 h) and Strep‐HRP (S000‐03, 1:500, Rockland, 1 h). Vimentin was visualized using DAB (ab64238, Abcam, 10 min). For cytokeratin, a second antigen retrieval was performed with pepsin (0.04% in 0.01 M HCl, 37°C, 10 min), followed by blocking (10% goat serum in blocking buffer, 15 min) and overnight incubation at 4°C with anti‐pan cytokeratin (NB600‐579, 1:75, Novus Biologicals). The next day, sections were kept at room temperature (2 h), and incubated with alkaline phosphatase–conjugated secondary antibody (ab6722, 1:500, Abcam, 1 h) and developed with NBT/BCIP (34 042, Thermo Scientific, 10 min) followed by Fast Red counterstaining (H‐3403, Vector Laboratories, 4 min). Slides were washed with Tris‐buffered saline containing 0.1% Tween‐20 (TBS‐T) between steps [79, 80].

Placental labyrinth compartments volumes (fetal capillaries, maternal blood spaces, and trophoblasts), surface areas (fetal capillaries and maternal blood spaces), fetal capillary measurements (length, area, and diameter), interhaemal barrier thickness, and theoretical and specific diffusion capacities were determined as previously described [57, 80].

### 4.7 Immunohistochemical Analysis of Metabolic Proteins in the Placenta and Maternal Pancreas

Primary antibodies used were insulin receptor β (INSRβ; 1:50, sc‐711), IGF1R (1:50, sc‐713), p‐mTOR (1:200, sc‐293 133), GLUT1 (1:100, sc‐377 228), and insulin (1:500, sc‐8033) (all Santa Cruz Biotechnology), and AKT (1:200, 9272S; Cell Signaling Technology Inc).

Immunohistochemistry was performed using the Dako EnVision FLEX, High pH (Link) polymer detection system (Agilent) as described by Santos et al. INSRβ, IGF1R, p‐mTOR, AKT, and GLUT1 protein abundance was assessed descriptively and quantitatively in placental junctional and labyrinth zones [24, 25]. Color deconvolution and thresholding were automated for all images using two custom ImageJ macros, ensuring consistent application of a predetermined threshold; scripts are available at https://github.com/ReisBia/ImageJ‐Scripts‐for‐IHC‐Stained‐Area‐Quantification.

In the maternal pancreas, insulin‐positive islets were quantified in three sections per sample, spaced 12 μm apart.

### 4.8 qRT‐PCR Analysis of Metabolic Genes in the Placenta

Total RNA was extracted from placental tissue using TRIzol reagent (15 596 026, Invitrogen). RNA was reverse transcribed using the GoTaq qPCR and RT‐qPCR Systems kit (A6010, PROMEGA). Target gene transcripts were quantified by qPCR using an Applied Biosystems 7500 Real‐Time PCR System (Thermo Scientific). All procedures were performed as reported by Santos et al. (2022b).

Primers for *Insr*, *Isr‐1*, *Igf1r*, *Igf1*, *mTor*, *Raptor*, *Glut1*, *rPrl*, *PlII* and *Leptin* were selected from previous studies or the National Center for Biotechnology Information based on *Rattus norvegicus* mRNA sequences (Table S4). Gene expression was calculated using the 2^−ΔΔCT^ method, normalized to *Polr2a* (stable across groups), and expressed relative to the control group [81, 82].

### 4.9 Statistical Analysis

Placental and fetal mass, placental efficiency, and fetal glycemia were analyzed using a mixed linear model with Bonferroni *post hoc* test. Mothers were included as subjects and random factors, treatment as a fixed factor, and litter size as a covariate (IBM SPSS Statistics for Windows, Version 23.0) [83]. Remaining data were analyzed by one‐ or two‐way ANOVA followed by Student–Newman–Keuls (SNK) test or Student's *t*‐test, as appropriate (GraphPad Prism 10.1.2). Data are presented as mean ± standard error of the mean (SEM), and differences were considered statistically significant at *p* < 0.05.

## Author Contributions

Conceptualization: B.R.S., J.F.S. Methodology: B.R.S., J.F.S., A.N.S.‐P., J.L.‐T. Investigation: B.R.S., J.M.A.C., L.C.S., C.S.O., M.C.P.S.A., N.P.R. Formal analysis: B.R.S. Validation: J.F.S. Visualization: B.R.S. Resources: J.F.S., R.S., A.N.S.‐P. Funding acquisition: J.F.S., J.L.‐T., A.N.S.‐P. Project administration: J.F.S. Supervision: J.F.S. Writing – original draft: B.R.S., J.F.S. Writing – review and editing: B.R.S., J.F.S., A.N.S.‐P., J.L.‐T. All authors read and approved the final version of the manuscript and contributed equally to the submitted work.

## Funding

This work was supported by the Brazilian research agencies Coordenação de Aperfeiçoamento de Pessoal de Nível Superior (CAPES) and Conselho Nacional de Desenvolvimento Científico e Tecnológico (CNPq; grant number 402515/2021‐8). J.L.‐T. is currently supported by an Attraction of Talent Grant from the Community of Madrid (grant number. 2023‐T1/SAL‐GL‐28960, CESAR NOMBELA fellowship). A.N.S.‐P. was funded by a Lister Institute of Preventative Medicine Research Grant (RG93692).

## Conflicts of Interest

The authors declare no conflicts of interest.

## Supporting information


**Figure S1:** Negative controls for each batch of immunohistochemistry markers.

**File S2: and S3:** Macro scripts used for automated ImageJ IHC (H–DAB) quantification, including color deconvolution, thresholding, and stained area measurement. Supplementary File_S2.txt performs automated color deconvolution across an entire image set, whereas Supplementary File_S3.txt performs automated thresholding and subsequent measurements across an entire image set.

**Table S4:** List of genes and nucleotide sequences for RT‐qPCR primers.

## Data Availability

The data that support the findings of this study are available from the corresponding author upon reasonable request.
