# Supplementary figures and images for: Kisspeptin Restores Placental mTOR Signaling and Improves Glucose Homeostasis Mediators Disrupted by Maternal Hypothyroidism in Rats
Source: Acta Physiol (Oxf). 2026 Mar 4;242(4):e70188. doi: 10.1111/apha.70188 (PMC12960837; doi:10.1111/apha.70188)

**Supplementary Figure**

**
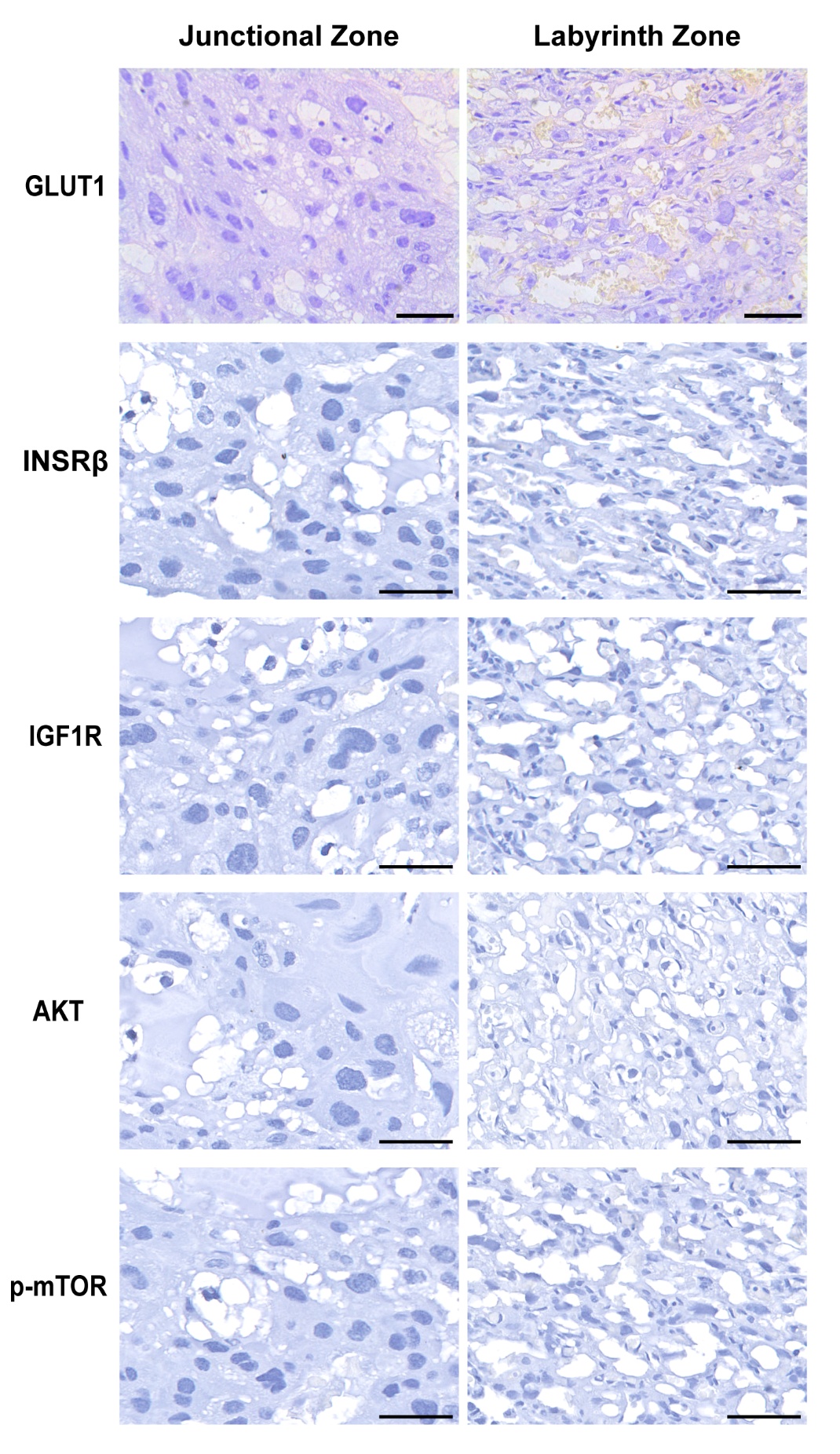
**

Supplement: Supplementary file 1 — Figure S1: Negative controls for each batch of immunohistochemistry markers. [file APHA-242-e70188-s001.docx]
