# Supplementary material for: Kisspeptin Restores Placental mTOR Signaling and Improves Glucose Homeostasis Mediators Disrupted by Maternal Hypothyroidism in Rats
Source: Acta Physiol (Oxf). 2026 Mar 4;242(4):e70188. doi: 10.1111/apha.70188 (PMC12960837; doi:10.1111/apha.70188)
Supplement: Supplementary file 3 — Table S4: List of genes and nucleotide sequences for RT‐qPCR primers. [file APHA-242-e70188-s003.docx]

**Supplementary Table**

Table 1 - List of genes and nucleotide sequences for RT-qPCR primers.

| **Gene** | **Sequence (5'->3')** | **References** |
| --- | --- | --- |
| *Insr* | F: GGCCCGATGCTGAGAACA | ^1^ |
|  | R: CGTCATTCCAAAGTCTCCGA |  |
| *Irs-1* | F: GGCACCATCTCAACAATC | ^2^ |
|  | R: GTTTCCCACCCACCATAC |  |
| *Igf1r* | F: CTGCTCCAAAGACAAAATACCCATC | NCBI |
|  | R: ACCGCACACTTCTGTCTTGG |  |
| *Igf1* | F: ACCCGGGACGTACCAAAATG | ^3^ |
|  | R: CGAGCTGGTAAAGGTGAGCA |  |
| *mTor* | F: GATACGCCGTCATTCCTC | ^4^ |
|  | R: TGCTCAAACACCTCCACC |  |
| *Raptor* | F: CCCTTTACACCATGCATAGCT | ^5^ |
|  | R: GAGGGTTACCATTGTGGAAGT |  |
| *Glut1(Slc2a1)* | F: CAATCAAACATGGAACCACCG | ^6^ |
|  | R: CGATTGATGAGCAGGAAGCG |  |
| *rPrl* | F: GCCTCTCAAGCTAAAGGACAC | NCBI |
|  | R: TTTTCTTCAGGTTGGCCCCTT |  |
| *PlII* | F: TTACCGAATGTCCACTGG | ^7^ |
|  | R: TGCAAATCTGACCACTCAG |  |
| *Lep* | F: GTTCCTGTGGCTTTGGTCCT | ^8^ |
|  | R: CTGGTGACAATGGTCTTGATGA |  |
| *Polr2a* | F: GCTGGACCTACTGGCATGTT | ^9^ |
|  | R: ACCATAGGCTGGAGTTGCAC |  |

NCBI = National Center for Biotechnology Information

**REFERENCES**

1 Haghir H, Rezaee A-A-R, Nomani H, Sankian M, Kheradmand H, Hami J. Sexual Dimorphism in Expression of Insulin and Insulin-Like Growth Factor-I Receptors in Developing Rat Cerebellum. *Cell Mol Neurobiol* 2013; **33**: 369–377.

2 Abdelmageed ME, Shehatou GSG, Suddek GM, Salem HA. Protocatechuic acid improves hepatic insulin resistance and restores vascular oxidative status in type-2 diabetic rats. *Environ Toxicol Pharmacol* 2021; **83**: 103577.

3 Santos BR, dos Anjos Cordeiro JM, Santos LC, Barbosa EM, Mendonça LD, Santos EO *et al.* Kisspeptin treatment improves fetal-placental development and blocks placental oxidative damage caused by maternal hypothyroidism in an experimental rat model. *Front Endocrinol (Lausanne)* 2022; **13**. doi:10.3389/fendo.2022.908240.

4 YIN L, LU L, LIN X, WANG X. Crucial role of androgen receptor in resistance and endurance trainings-induced muscle hypertrophy through IGF-1/IGF-1R- PI3K/Akt- mTOR pathway. *Nutr Metab (Lond)* 2020; **17**: 26.

5 Mazumder AG, Patial V, Singh D. Mycophenolate mofetil contributes to downregulation of the hippocampal interleukin type 2 and 1β mediated PI3K/AKT/mTOR pathway hyperactivation and attenuates neurobehavioral comorbidities in a rat model of temporal lobe epilepsy. *Brain Behav Immun* 2019; **75**: 84–93.

6 Guo J, Fang M, Zhuang S, Qiao Y, Huang W, Gong Q *et al.* Prenatal dexamethasone exposure exerts sex-specific effect on placental oxygen and nutrient transport ascribed to the differential expression of IGF2. *Ann Transl Med* 2020; **8**: 233–233.

7 Lee CK, Moon DH, Shin CS, Kim H, Yoon YD, Kang HS *et al.* Circadian expression of Mel1a and PL-II genes in placenta: effects of melatonin on the PL-II gene expression in the rat placenta. *Mol Cell Endocrinol* 2003; **200**: 57–66.

8 Lecoutre S, Oger F, Pourpe C, Butruille L, Marousez L, Dickes-Coopman A *et al.* Maternal obesity programs increased leptin gene expression in rat male offspring via epigenetic modifications in a depot-specific manner. *Mol Metab* 2017; **6**: 922–930.

9 Santos BR, dos Anjos Cordeiro JM, Santos LC, de Oliveira LS, Mendonça LD, Santos EO *et al.* Maternal hypothyroidism reduces the expression of the kisspeptin/Kiss1r system in the maternal-fetal interface of rats. *Reprod Biol* 2022; **22**: 100615.
